# Cardiometabolic Index (CMI), Lipid Accumulation Products (LAP), Waist Triglyceride Index (WTI) and the risk of acute pancreatitis: a prospective study in adults of North China

**DOI:** 10.1186/s12944-023-01948-3

**Published:** 2023-11-09

**Authors:** Qiu Sun, Qingshuai Ren, Liming Du, Shuohua Chen, Shouling Wu, Bing Zhang, Bangmao Wang

**Affiliations:** 1 Department of Gastroenterology and Hepatology, Tianjin Medical University General Hospital, Tianjin Institute of Digestive Diseases, Tianjin Key Laboratory of Digestive Diseases, 154 Anshan Road, Heping District, Tianjin, 300052 China; 2 Department of Hepatobiliary, Kailuan General Hospital, No.57 Xinhua East Street, Tangshan, 063000 China; 3 Deparment of Cardiovascular Surgery, North China University of Science and Technology, Tangshan, 063000 China; 4 Department of Cardiology, Kailuan General Hospital, North China University of Science and Technology, No.57 Xinhua East Street, Tangshan, 063000 China

**Keywords:** Acute pancreatitis, Anthropometry visceral adiposity index, Cardiometabolic index, Lipid accumulation products, Waist triglyceride index, Prospective cohort study

## Abstract

**Objective:**

To investigate the correlation between anthropometric indexes [cardiometabolic index (CMI), lipid accumulation products (LAP), waist triglyceride index (WTI), and body mass index (BMI)] and acute pancreatitis (AP) in a Chinese adult population.

**Methodology:**

The present investigation consisted of a prospective group including 117,326 subjects who were enrolled in the Kailuan investigation. The individuals were categorized into quartiles based on their baseline levels of CMI, LAP, and WIT. BMI was categorized into three distinctive groups: normal weight group (BMI < 24 kg/m^2^), overweight group (BMI 24–28 kg /m^2^), and obesity group (BMI ≥ 28 kg/m^2^). The data were subjected to analysis in order to investigate the correlation between these anthropometric indexes and the incidence of AP. Cox regression models were employed to assess the relative risk of AP while accounting for known risk factors through appropriate adjustments.

**Outcomes:**

Over the course of a median follow-up duration of 12.59 ± 0.98 years, we documented 401 incident AP cases. Incidence density and cumulative incidence rates of AP increased with the increase of CMI, LAP, and WTI. After multivariate adjustment, the fourth quartile of CMI, LAP, and WTI exhibited the greatest risk of AP [CMI: hazard ratio (HR) 1.93, 95% confidential interval (CI) (1.45–2.57); LAP: HR 2.00, 95% CI(1.49–2.68); WTI: HR 2.13,95% CI (1.59–2.83)]. In comparison to the normal weight group, the obesity group (BMI ≥ 28 kg/m^2^) had an elevated risk of AP (HR = 1.58, 95% CI: 1.21–2.05). Furthermore, the incremental effect of BMI combined with CMI on the prognostic value of AP was greater than that of BMI alone (the C statistics demonstrated a result of 0.607 versus 0.546; the integrated discrimination improvement revealed a result of 0.321%; net reclassification improvement was 1.975%).

**Conclusion:**

We found that CMI, LAP, and WTI were positively and independently connected to the risk of AP. Additionally, CMI demonstrates a superior prognostic capacity than other indexes in anticipating AP.

**Supplementary Information:**

The online version contains supplementary material available at 10.1186/s12944-023-01948-3.

## Introduction

Acute pancreatitis (AP) is considered the greatest prevalent gastrointestinal disease. The annual occurrence of AP in high-income nations is reported to be 34 occurrences per 100,000 person-years [1], and the fatality rate varies from 2 to 10% [2]. In population studies from different countries over the last 20 years, the occurrence of the first AP infection was 15–45 cases / 100 000 person-years, the occurrence of chronic pancreatitis was 4–10 cases / 100 000 person-years, and the prevalence was 13–52 cases / 100 000 person-years [3]. AP is distinguished by both localized and systemic inflammatory responses and has a fluctuating clinical outcome. The majority of individuals exhibit mild AP, a condition that is self-limited. Around 20% of individuals experience the development of moderate to serious AP, characterized by necrosis of the pancreas or peripancreatic tissues, as well as organ failure or a combination of both [4, 5]. A meta-analysis conducted on population-based cohort studies demonstrated that the worldwide occurrence and fatality rates of AP were found to be 33.74/100,000 and 1.60/100,000, respectively [1]. With the increasing occurrence and decreasing fatality of AP, its prevalence within the population and the reasons for hospitalization have increased, bringing high costs to the healthcare system [6].

The connection between BMI and the incidence of AP has been validated by prospective cohort studies undertaken in Western populations [7, 8]. A positive association between AP and BMI in older women was revealed in an investigation conducted in the United States, which documented a total of 660 cases of acute or chronic pancreatitis [7]. The Swedish Mammography Cohort (SMC) and The Cohort of Swedish Men (COSM), which incorporated more than 1000 instances of AP, utilized objective measures instead of self-reported BMI, showing a favorable dose–response correlation of BMI with the incidence of AP was higher than earlier investigations in Western populations [8]. BMI is widely employed as a prevalent metric for evaluating obesity. However, it possesses certain constraints when it comes to evaluating the distribution of body fat [9]. BMI measurements do not distinguish between trunk and visceral obesity, whereas anatomical fat distribution is considered important because it produces different metabolic effects. Therefore, we should pay special attention to visceral adipose tissue, that is, the increase of adipose tissue around the organs in the abdominal cavity [10]. At present, some new visceral adiposity indexes (cardiometabolic index (CMI), lipid accumulation product (LAP), and waist triglyceride index (WTI)) are closely related to metabolic syndrome (MetS) [11]. LAP, which combines triglycerides (TG) and waist circumference (WC) and is set for sex, is considered to provide a beneficial assessment of visceral adipose tissue and has a better correlation with metabolic conditions [12]. CMI is a novel index that has been developed based on the values of TG/HDL-C and WHtR. This index can be conveniently obtained during a routine health checkup [13]. Several studies have shown that CMI is firmly correlated with obesity-associated metabolic disorders, including diabetes and cardiovascular disorders (CVD) [10, 14, 15]. Several studies have shown that LAP is a robust anthropometric marker for predicting insulin resistance (IR), diabetes, MetS, and nonalcoholic fatty liver disorder (NAFLD) [16–19]. Inspired by the TyG index formula, Liu et al. integrated WC with TG to establish a novel index, namely WTI, which demonstrated a robust capacity to characterize MetS [20]. As far as we know, CMI, LAP, and WTI have been confirmed to exhibit associations with diverse metabolic disorders, but the correlation with AP is rarely reported, especially the study of CMI and the risk of AP has not been reported, and whether CMI, LAP, and WTI provide higher predictive value for AP than BMI is also unknown. Therefore, a prospective cohort study has been performed using the data of Kailuan study population to evaluate the impact of traditional measures of BMI and new measures of CMI, LAP, and WTI on the incidence of AP so as to provide a reference and basis for research in the field of pancreatitis.

## Methods

### Study participants

The Kailuan Study is a multicenter, community-based prospective cohort study located in Tangshan, an industrial city in China, which was established by Kailuan General Hospital and eleven affiliated hospitals, as detailed elsewhere [21]. Subjects from the Kailuan community were recruited and finished the first survey, such as questionnaire, physical examination, and laboratory assessment, from 2006 to 2009, and were followed up every two years. A total of 126,847 employees and retirees from the Kailan Group were included in the first health experience, and 117,326 employees who satisfied the specified criteria were incorporated into the present investigation.

Inclusion criteria: (1) employees and retirees of Kailuan Group who participated in the first physical examination from 2006 to 2009; (2) no cognitive impairment, able to complete the questionnaire; (3) no serious physical defects; (4) Complete data of traditional AP risk factors such as alcohol consumption, physical exercise, history of hypertension and diabetes, and routine physical and chemical indicators.Exclusion criteria: (1)previous history of pancreatitis;(2)pancreatitis caused by other risk factors, such as pancreatitis-inducing drugs or recent invasive procedures such as ERCP; (3) baseline data of height, weight, WC, TG, and high-density lipoprotein were missing. (4) baseline WC, height, weight, TG, and high-density lipoprotein extreme data. After excluding 8248 cases with missing data (height, weight, WC, TG, and high-density lipoprotein), 1255 cases with abnormal values (height, weight, WC, TG, and high-density lipoprotein), and 18 cases with previous history of pancreatitis. There were no cases of pancreatitis due to recent invasive procedures, such as ERCP, and there were no data for drug-induced pancreatitis.117326 instances were finally incorporated within the statistical analysis, of which 401 cases were with AP.

### Standard protocol approvals, registrations and patient consent

The research was granted approval by the Ethics Committee at Kailuan General Hospital. The Kailuan study was registered at the International Clinical Trials Registry Platform (apps.who.int/trialsearch/Trial2.aspx?TrialID = ChiCTR-TNRC-11001489) with research identifying number ChiCTR-TNRC-11001489.

### AP assessment

AP is characterized by the manifestation of mild, moderate, or severe AP incidents within the designated observation period in the absence of any prior medical record indicating a history of AP. The diagnosis was made according to the American Gastroenterological Association Institute Guideline on Initial Management of AP diagnostic criteria [22]. Between June 2006 and October 2007, individuals from the Kailuan community were recruited as participants and successfully finished the initial survey, which consisted of questionnaires, physical examinations, and laboratory evaluations. Subsequently, a follow-up was conducted every two years. The follow-up began at the end of the first physical examination, and the end of follow-up was AP events, death, loss of follow-up, and the end of study (January 01, 2020). More than two episodes of AP were counted as one AP event, and the outcome was defined as the time and event that first occurred in AP. The study participants in the Kailuan group were connected to the Municipal Social Insurance Institution database and Hospital Discharge Register in order to detect instances of AP. This comprehensive approach ensured that all cases within the group were accounted for. The identification of instances of AP was facilitated through the utilization of ICD-9 and ICD-10. The Kailuan research has been collecting supplementary data on the medical history of AP through biennial questionnaires since 2006. Data regarding mortality was obtained from regional vital statistics offices. Three physicians conducted a thorough examination of the medical records for cases of AP that were identified using the ICD code or a questionnaire.

### Assessment of variables

Information on birth date, sex, smoking status, alcohol consumption, physical exercise, and past medical history (hypertension, diabetes, and lipid-lowering medications, etc.) were collected via questionnaires administered by the research doctors at the baseline interview. Smoking status was stratified into 2 levels: Smokers were defined as ≥ 1 cigarette per day for more than 1 year, and the rest were non-smokers. Drinking status was stratified into 2 levels: The drinkers were those who drank liquor (alcohol content above 50%) ≥ 100 ml per day on average for more than 1 year, and the rest were non-drinkers. Physical exercise was defined as exercising ≥ 3 times per week for ≥ 30 min each time. Hypertension was defined as the presence of any of the following: a history of hypertension, current treatment to lower blood pressure, a systolic blood pressure of 140 mmHg or higher, or a diastolic blood pressure of 90 mmHg or higher. Diabetes diagnosis criteria are as follows: Have a history of diabetes, are taking insulin or oral hypoglycemic agents, or fasting blood glucose level ≥ 7.0 mmol/L. Heart rate, height, weight, and waist circumference were measured by trained nurses and body mass index was calculated as weight in kilograms divided by height in meters squared. Blood samples were obtained from the antecubital vein and transfused into vacuum tubes containing EDTA in the morning after an overnight fasting period. Within 30 min of collection, the blood was centrifuged for 10 min at 3000 rotations per minute at 25 °C. An auto analyzer (Hitachi 747; Hitachi, Tokyo, Japan) was used to measure fasting blood glucose(FBG),total cholesterol(TC),triglyceride(TG), high-density lipoprotein cholesterol (HDL-C),and low-density lipoprotein(LDL-C) at the central laboratory of Kailuan hospital.

### Anthropometric indices calculations

BMI was assessed by calculating the ratio of an individual's weight in kilos to the square of their height in meters. The waist-to-height ratio (WHtR) is calculated by dividing WC by height, both measured in centimeters.

The anthropometric indices were computed using the subsequent formulas:

$$\begin{aligned} &\text{CMI=TG(mmol/L)/HDL-C(mmol/L)}^{*}\text{WHtR};\\&\text{LAP(females)=TG(mmol/L)}^{*}\! \left[\text{WC(cm)}\!-\!58\right]\!;\\ &\text{LAP(males)=TG(mmol/L)}^{*}\!\left[\text{WC(cm)}\!-\!65\right]\!;\\ &\text{WTI=WC(cm)}^{*}\text{TG(mmol/L)}.\end{aligned} $$

### Statistical analysis

The data input process involved the submission of information by a designated data entry clerk at each individual hospital. Subsequently, this data was uploaded to the Oracle10.2 database, which is situated at Kailuan Hospital, utilizing the Internet. The data have been subjected to evaluation utilizing SAS 9.4. The measurement data, which followed a normal distribution, were represented as (x ± s). A one-way analysis of variance was performed to evaluate the comparison between groups. The measurement data, which exhibited a skewed distribution, were denoted as M (P25 ~ P75). To compare across groups, a nonparametric test was employed for analysis. The count data were represented as relative numbers, and the contrast between groups was evaluated utilizing the chi-square test. The incidence density was calculated utilizing the following formula: the number of AP cases during the observation period × 10 000/ the total follow-up time of observation subjects during the observation period. Kaplan–Meier technique was utilized for calculating the cumulative occurrence of AP within distinct groups, and the Log-Rank test was employed for comparing the cumulative occurrence between the different groups. The survival curve was drawn by the Kaplan–Meier technique and tested by the Log-Rank assay. COX proportional hazard regression model was used to calculate the HR values (95% CI) between the quartile groups of LAPS, WTI, and CMI and new cases of AP. The nomogram was conducted by R software (version 4.2.3). Furthermore, we employed C statistics, combined discrimination enhancement, and net reclassification index as metrics to assess and compare the incremental prognostic value of LAP, WTI, CMI, and BMI impact. All statistical tests conducted in the study were two-sided, and *P* < 0.05 was reported as statistical significance.

## Results

### Baseline features

From 2006 to 2009, 126,847 subjects from the Kailuan community were included in the first health experience. Following a sequence of exclusion criteria, a total of 117326 participants were incorporated within the current investigation (Additional file 1: Figure S1). Table 1 shows the clinical and laboratory features at baseline based on the quartiles of CMI level. In contrast to those within the lowest quartile group, individuals exhibiting a greater CMI have been shown to possess certain characteristics such as advanced age, male gender, increased obesity as indicated by higher BMI and WC, and a higher prevalence of AP among participants. Similarly, significant variations in biological characteristics were noted across the groups. FPG, TG, and LDL-C levels of the subjects within the highest quartile of CMI were found to be considerably elevated compared to those in the first quartile. Furthermore, it was shown that the groups with elevated CMI levels exhibited a greater incidence of comorbidities, including diabetes,hypertension,and cholelithiasis.

**Table 1:** Baseline characteristics of participants according to quartiles of CMI

Characteristics	Quartiles of CMI				*F(*× *2)*	*P* Value
	G1 (≤ 0.29)	G2 (0.29–0.45)	G3 (0.45–0.73)	G4 (≥ 0.73)		
Number	29637	29655	28545	29489		
No. Of AP patient	72	97	88	144		
Age, year	50.04 ± 13.71	50.96 ± 12.94	52.10 ± 12.62	51.47 ± 11.78	116.87	< 0.001
Men, n(%)	22110 (74.60)	23746 (80.07)	23411 (82.01)	25125 (85.20)	805.32	< 0.001
BMI,kg/m2	23.03 ± 3.02	24.58 ± 3.11	25.70 ± 3.20	26.72 ± 3.28	5397.63	< 0.001
WC,cm	81.18 ± 8.89	85.62 ± 8.57	88.94 ± 8.80	91.87 ± 8.96	6148.98	< 0.001
Ht,cm	167.11 ± 6.91	167.70 ± 6.82	167.77 ± 6.90	168.12 ± 6.80	82.02	< 0.001
FBG,mmol/L	5.19 ± 1.31	5.38 ± 1.48	5.57 ± 1.69	5.84 ± 2.09	601.24	< 0.001
TG,mmol/L	0.74 ± 0.22	1.11 ± 0.27	1.55 ± 0.41	3.20 ± 1.71	33130.40	< 0.001
TC,mmol/L	4.80 ± 0.97	4.94 ± 1.01	5.06 ± 1.11	4.96 ± 1.44	177.23	< 0.001
HDL-C,mmol/L	1.73 ± 0.41	1.55 ± 0.34	1.45 ± 0.33	1.35 ± 0.35	4312.59	< 0.001
LDL-C,mmol/L	2.30 ± 0.95	2.46 ± 0.90	2.50 ± 0.92	2.36 ± 1.05	205.57	< 0.001
Drinker, n(%)	12245 (41.32)	11519 (38.84)	12190 (42.70)	13634(46.23)	278.13	< 0.001
Smoker, n(%)	11523 (38.88)	11315 (38.16)	12119 (42.46)	13687 (46.41)	391.36	< 0.001
Physical exercise, n(%)	4706 (15.88)	4633 (15.62)	4808 (16.84)	4922(16.69)	14.29	< 0.001
Hypertension, n (%)	9302 (31.39)	12511 (42.19)	13704 (48.01)	16147 (54.76)	2548.35	< 0.001
Diabetes, n(%)	1364 (4.60)	2146 (7.24)	2915 (10.21)	4357(14.78)	1359.06	< 0.001
Cholelithiasis, n(%)	829 (2.80)	932 (3.14)	1075 (3.77)	1043 (3.54)	1.09	< 0.001
Education ≥ 9 years, n(%)	7537(25.43)	5942 (20.04)	5982 (20.96)	6219(21.09)	326.29	< 0.001
Using lipid lowering drugs, n(%)	155 (0.52)	226 (0.76)	328 (1.15)	557 (1.89)	296.24	< 0.001

Abbreviations: *AP* Acute pancreatitis, *BMI* Body mass index, *WC* Waist circumference, *Ht* Height, *FBG* Fasting blood glucose, *TC* Total cholesterol, *TG* Triglyceride, *HDL* High-density lipoprotein, *LDL* Low-density lipoprotein

CMI = TG(mmol/L)/HDL-C(mmol/L)*WHtR

### The occurrence of AP in the quartile groups of CMI

**Fig. 1:**
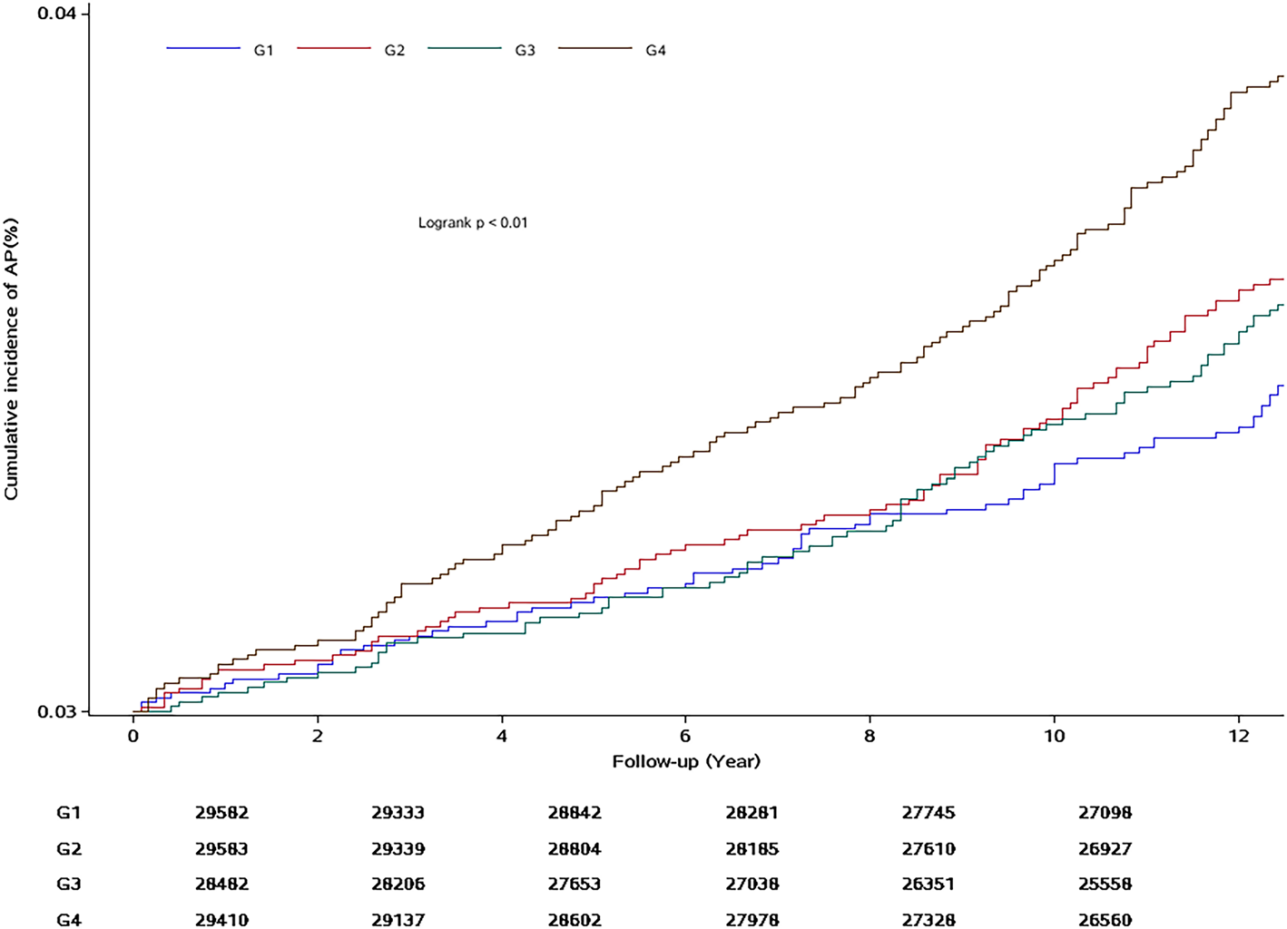
The cumulative incidence rates in the quartile groups of CMI level. CMI = TG(mmol/L)/HDL-C(mmol/L)*WHtR; G1 = CMI ≤ 0.29;G2 = 0.29 < CMI ≤ 0.45;G3 = 0.45 < CMI < 0.73;G4 = CMI ≥ 0.73

Over the course of a mean follow-up period of (12.59 ± 0.98) years, 401 cases of AP occurred, with 72,97,88,144 new cases of AP in each CMI quartile group, and the incidence densities were 1.75, 2.37, 2.25, and 3.55/10 000 person-years, respectively. Incidence density and cumulative incidence rates of AP increased with CMI. Figure 1 presents the cumulative incidence rates in the quartile groups of CMI level.

### Association of CMI, LAP, WTI and BMI with the risk of AP

**Fig. 2:**
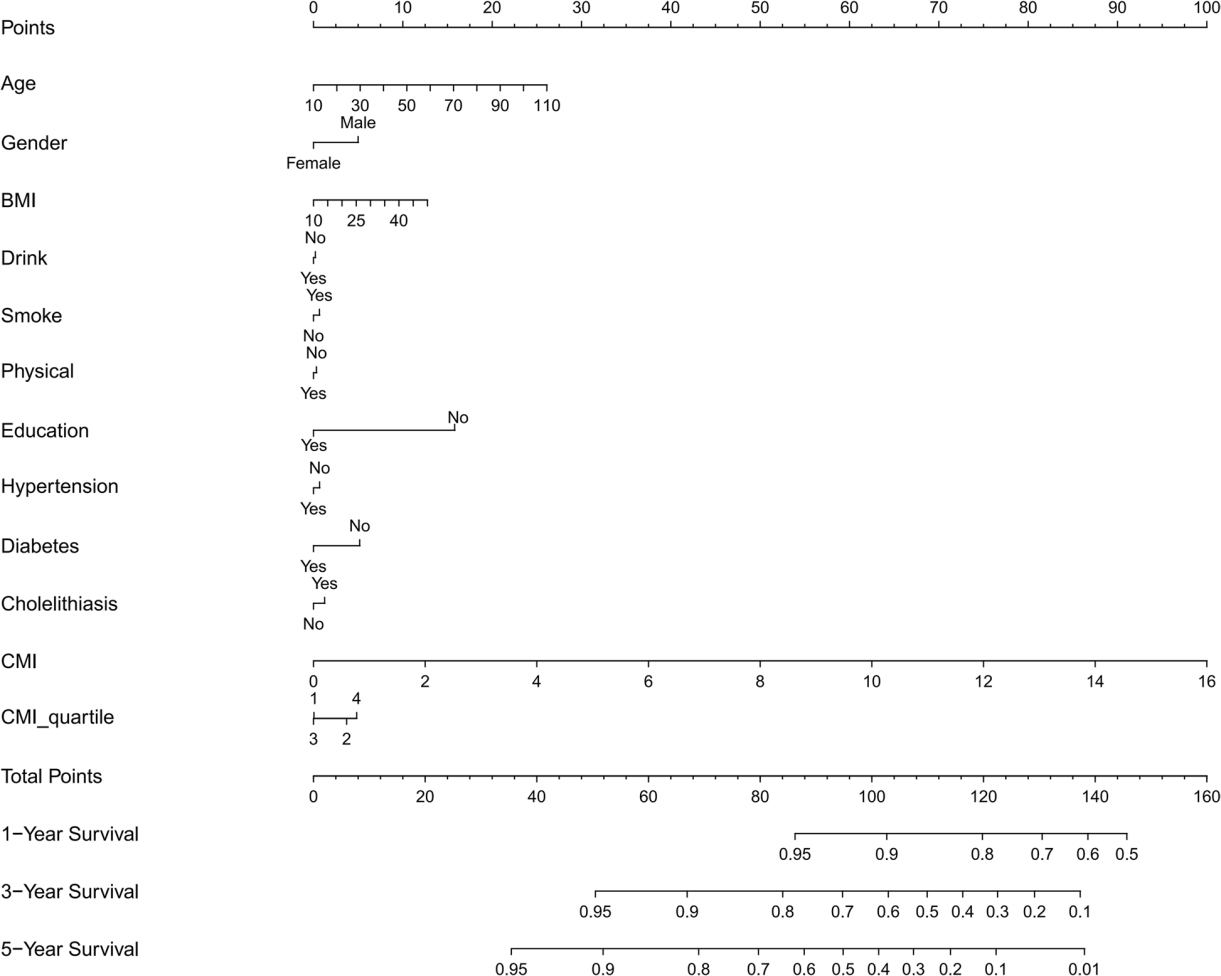
Construction of a nomogram based on the multivariate Cox regression analysis of CMI. Abbreviations: CMI, Cardiometabolic Index; BMI = body mass index

Baseline CMI, LAP, and WTI quartile groups have been evaluated via COX proportional hazards regression model with AP, and Table 2 presents the results. Taking the lowest quartile of CMI, LAP, and WTI as the reference without adjusting for other confounding factors, the results showed that the HRs (95% CIs) within the second, third, and greatest quartile of CMI have been 1.35(0.99–1.83), 1.29(0.94–1.76), and 2.03(1.53–2.69), respectively. The HRs (95% CIs) within the second, third, and greatest quartile of LAP were 1.26 (0.92–1.73), 1.41 (1.04–1.92), and 2.14 (1.61–2.85), respectively. The HRs (95% CIs) in the second, third, and highest quartile of WTI have been 1.14 (0.83–1.57), 1.41 (1.04–1.92), and 2.24 (1.69–2.97), independently. According to the BMI classification standard defined by "Chinese Expert Consensus on Medical nutrition treatment of overweight/obesity" [23], the observation population was separated into the normal weight group (BMI < 24 kg/m^2^), overweight group (BMI 24–28 kg /m^2^), and obesity group (BMI ≥ 28 kg/m^2^). Taking the Normal weight group as a reference, the adjusted HRs (95% CIs) within the overweight group and obese group BMI have been 1.14 (0.91–1.43) and 1.64(1.27–2.12). The highest CMI, LAP, and WTI quartiles were associated with the highest risk for AP. Results were comparable within an age-,sex- and BMI-adjusted model and in a multivariable-adjusted model. According to the relevant factors of the multivariate cox regression analysis of CMI, the nomogram was constructed (Fig. 2).

**Table 2:**
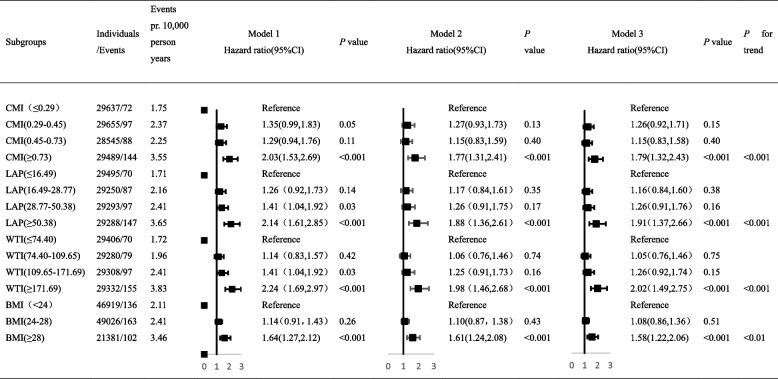
Association of CMI, LAP, WTI and BMI with Acute pancreatitis in Cox proportional hazard models

Abbreviations: *CMI* Cardiometabolic Index, *LAP* Lipid accumulation product, *WTI* Waist-triglyceride index, *HR* Hazard ratio, *CI* Confidence interval

Model 1,unadjsted;

Model 2, adjusted for age,sex and BMI;

Model 3,further adjusted for variables in model 2 plus smoking, alcohol drinking, physical activity, diabetes, hypertension,cholelithiasis,education and using lipid lowering drugs

CMI = TG(mmol/L)/HDL-C(mmol/L)*WHtR

LAP(females) = TG(mmol/L)*[WC(cm) − 58];LAP(males) = TG(mmol/L)*[WC(cm) − 65]

WTI = WC(cm)*TG(mmol/L)

Stratified by age, using the lowest quartile of CMI, LAP and WTI as controls, the HRs (95% CIs) of AP in age ≤ 40 years,after adjusting for multivariable factors, the highest quartile of CMI, LAP and WTI were 5.94 (2.42,14.55), 6.41(2.68,15.34), and 7.20(2.89,17.90), respectively. The HRs (95% CIs) of AP in the highest quartile of CMI, LAP, and WTI were 1.62(1.08,2.42), 1.73(1.13,2.64), and 1.96(1.32,2.93) in the 40–60 years group, respectively. The HRs (95% CIs) of AP in age ≥ 60 years, the highest quartile of CMI, LAP, and WTI were 1.15(0.63,2.09), 1.19(0.62,2.32), and 1.11(0.61,2.04), respectively.In the normal weight group (BMI < 24 kg/m2) as the control group, age ≤ 40 years, after adjusting for multivariable factors, the HR(95% CIs) of AP occurrence in the obesity group (BMI ≥ 28 kg/m2) was 3.21(1.64,6.30). When the age was between 40 and 60 years, the HR(95% CIs) of AP in the obesity group was 1.37(0.96,1.94). The HR(95% CIs) of AP in the obesity group was 1.42(0.84,2.40) when the age was ≥ 60 years.Stratified by gender, the HRs (95% CIs) of AP in the highest quartile of CMI, LAP, WTI and obesity group were 2.50(1.04,6.02), 2.25(0.85,5.94), 3.76(1.50,9.40), and 1.81(0.94,3.50), respectively.The HRs (95% CIs) of AP in men with the highest quartile of CMI, LAP, WTI and obesity group were 1.70(1.23,2.36), 1.93(1.36,2.74), 1.86(1.34,2.57) and 1.55(1.16,2.07), respectively.The results are detailed in Table 3.

**Table 3:**
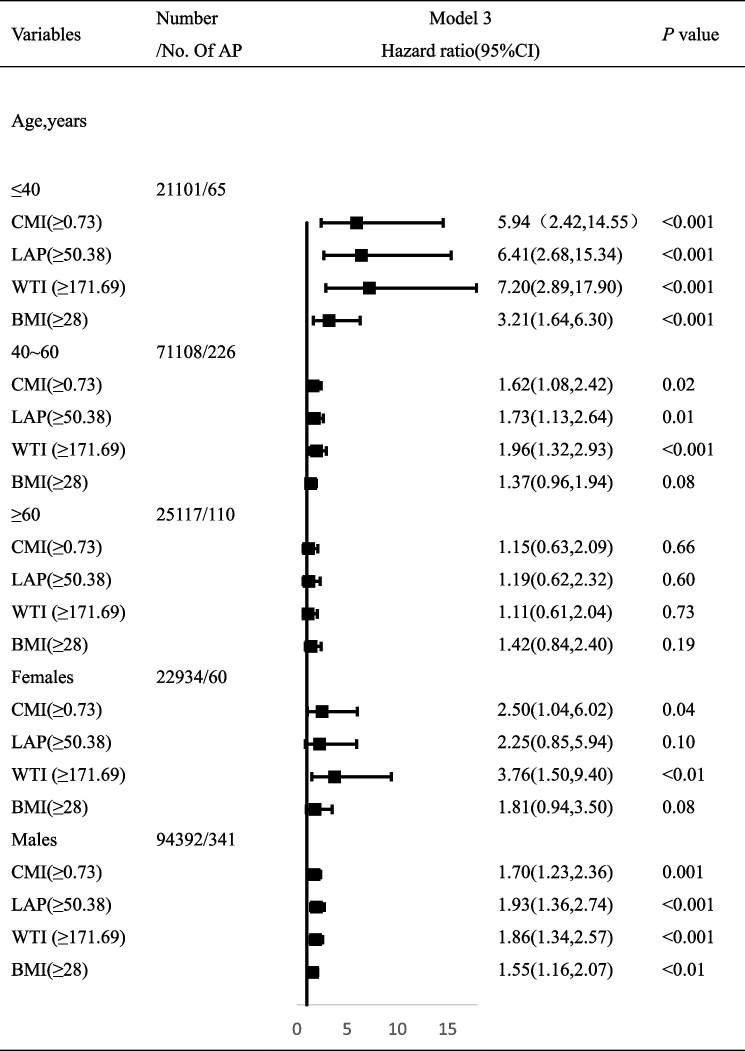
Adjusted HRs (95% CIs) for risk of acute pancreatitis according to sex—and age-specific CMI, LAP, WTI, and BMI

Abbreviations: *AP* Acute pancreatitis, *CMI* Cardiometabolic Index, *LAP* Lipid accumulation product, *WTI* Waist-triglyceride index, *HR* Hazard ratio, *CI* Confidence interval

### Incremental predictive value of BMI and CMI, LAP and WTI in AP

We conducted a comparative analysis of the prognostic value of BMI and CMI, LAP, and WTI in AP (Table 4). The C-statistics related to BMI exhibit enhancement upon the inclusion of CMI (from 0.546 to 0.607, *P* < 0.001), LAP (from 0.546 to 0.597, *P* < 0.001), and WTI (from 0.546 to 0.593,* P* < 0.001), respectively. The discriminatory capacity and risk reclassification of CMI, LAP, and WTI were shown to be significantly superior to BMI. The combined discrimination enhancement for CMI, LAP, and WTI was 0.321% (95% CI, 0.290%–0.351%; *P* < 0.0001), 0.318% (95% CI, 0.289%–0.349%; *P* < 0.0001), 0.302% (95% CI, 0.271%–0.333%; *P* < 0.0001), independently. The corresponding net reclassification index for CMI, LAP, and WTI was 1.975% (95% CI, 1.953%–1.997%; *P* < 0.0001), 1.935% (95% CI, 1.901%–1.969%; *P* < 0.0001), and 1.930% (95% CI, 1.894%–1.966%; *P* < 0.0001), independently. Among them, the inclusion of integrated impact of BMI and CMI can significantly also enhance the C statistics, the discriminatory capacity, and risk reclassification. This result provides substantial proof supporting the assertion that the incremental effect of CMI, LAP, and WTI on the predictive value of AP is higher than that of BMI, especially CMI.

**Table 4:** Reclassification and discrimination statistics for BMI and CMI, LAP, WTI predictive value in acute pancreatitis

	C statistics		IDI		NRI	
	Estimate (95% CI)	*P* value	Estimate (95% CI)	*P* value	Estimate (95% CI)	*P* value
BMI(Reference)	0.546 (0.512,0.580)		Reference		Reference	
CMI	0.607 (0.572,0.642)	< 0.001	0.321 (0.290,0.351)	< 0.001	1.975 (1.953,1.997)	< 0.001
LAP	0.597 (0.562,0.632)	< 0.001	0.318 (0.289,0.349)	< 0.001	1.935 (1.901,1.969)	< 0.001
WTI	0.593 (0.558,0.627)	< 0.001	0.302 (0.271,0.333)	< 0.001	1.930 (1.894,1.966)	< 0.001

Abbreviations: *CMI* Cardiometabolic Index, *LAP* Lipid accumulation product, *WTI* Waist-triglyceride index, *BMI* Body mass index, *IDI* Integrated discrimination improvement, *NRI* Net reclassification index

## Discussion

One notable advantage of this report is the considerable extent of sample size, which is 126,847 participants from the Kailuan community. In this study, we again confirmed that BMI was positively associated with AP. In addition, and more importantly, we identified a significant association of the new measures of CMI, LAP, and WTI with the incidence of AP. Based on our present comprehension, this investigation presents the initial evidence that directly demonstrates a significant correlation between CMI, LAP, and WTI and the prevalence of AP.

Based on the Kailuan cohort study, it was shown that there was a positive association rate between BMI and the risk of developing AP. In comparison to the normal weight group, the risk of AP within the obese group (BMI ≥ 28 kg/m^2^) was significantly higher, which aligned with the findings of earlier research investigations [24–26]. Hansen et al. [27] performed a prospective investigation on a cohort of 118,085 participants enrolled in Copenhagen, Denmark, while Prizment et al. [7] performed a prospective investigation on a cohort of 36,436 elderly women > 65 years old in Iowa, USA. Both found that BMI ≥ 25 kg/m^2^ has been correlated with a greater risk of AP. Recently, Choi et al. [28] found an elevated risk of AP when BMI > 27.5 kg/m^2^ in a prospective study of about 513,000 Koreans. These findings all support that overweight/obesity consider a risk factor in the context of AP. Compared to the existing literature, this study has a wider age range. Thus the results are more reliable. Obesity is considered to be chronic low-grade inflammation, which not only can elevate AP's risk of incidence but is also a risk factor for poor prognosis of AP [29]. The precise contribution of obesity to the development of AP remains incompletely understood, potentially due to the subsequent factors: first, the elevation of BMI will result in the increase of pancreatic fat content, which has direct biological toxicity to the pancreatic tissue itself [24]. Second, lipolysis releases a large amount of free fatty acids, which can directly damage pancreatic acinar cells and vascular endothelial cells, resulting in the formation of a local ischemic, hypoxic, and acidic environment of the pancreas, accelerating the activation of trypsinogen and aggravating the self-digestion and damage of pancreas [25]. In addition, obesity can lead to the imbalance of adipokines, causing inflammation, metabolic disorders, and multi-target organ damage [26].

Recent evidence suggests that simple, inexpensive anthropometric measurements have the potential to be used for subsequent diagnosis of obese individuals at increased risk of developing MetS. Central obesity is a condition that is closely linked to an individual's health status. In addition to central obesity, various other methods have been suggested for evaluating abdominal adipose tissue. These methods include measuring hip circumference (HC), WC, waist-to-hip ratio (WHR), WHtR, body shape index (ABSI), body adiposity index (BAI), body roundness index (BRI), and BMI [30]. However, these measures still cannot accurately distinguish visceral from subcutaneous adipose tissue, so the CMI, LAP, and WTI were presented to assess visceral fat distribution. Multiple investigations have indicated that these obesity indexes can contribute to a more precise evaluation of MetS, such as hyperlipidemia, hyperuricemia, and diabetes [31, 32]. Since 2015, CMI has been suggested as a new indicator for assessing visceral obesity utilizing lipid indicators and WHtR [13]. There is evidence to suggest that CMI is linked to a range of metabolic disorders, and it also demonstrates a greater capacity for predicting the occurrence of hyperuricemia [30], NAFLD [10], and CVD [33] in a random population compared to other traditional anthropometric measures. LAP, devised for the U.S. National Health and Nutrition Examination Survey, was employed as an indicator of central obesity [34]. LAP is demonstrated to be correlated with IR, prediabetes/diabetes [35], hyperuricemia [15], MetS [11], cardiovascular disease [36], and NAFLD [37]. Chinese study revealed WTI had been a positive prognostic value of MetS in both males and females [38]. Furthermore, it should be noted that WTI has been identified as a potential indicator for the development of coronary artery disorders [39] and in the occurrence of Metabolic dysfunction-associated fatty liver disease (MAFLD) [40].

Throughout our investigation, we evaluated the association of three low-cost, non-invasive, and simply computed anthropometric measures, including CMI, LAP, and WTI, with the potential risk of AP. We found that greater values of CMI, LAP, and WTI not only increased the incidence density and occurrence of AP but also increased the incidence rate of AP after adjusting for other relevant confounders. We found that CMI, LAP, and WTI have better predictive ability than BMI for AP. Obesity is divided into subcutaneous fat and visceral fat. The larger the area of visceral fat, the more likely it is to develop AP, the more chances it will develop local or systemic complications, and the more related it is to the occurrence of diabetes after AP [41]. Therefore, BMI was weaker than CMI, LAP, and WTI in predicting AP. In accordance with the NHANES III survey in the US, Kahn et al. revealed that individuals with elevated WC and TG had greater levels of fasting insulin and fasting plasma glucose than those with normal WC and TG. Metabolic abnormalities might arise as a result of elevated lipid accumulation, as shown by WC and TG levels [42]. LAP and WTI are composed of WC and TG. Therefore, they have a better ability to predict AP than BMI, whereas LAP, calculated according to sex, has a better predictive ability than WTI. CMI is relied on TG/HDL-C and WHtR values and contains not only WC and TG but also height and HDL-C.TG is the lipid component with the highest content and largest capacity in the body, while HDL-C can transport extrahepatic cholesterol into the liver, which is conducive to reducing blood lipids and anti-atherosclerosis. Therefore, CMI combined with BMI is more valuable than LAP and WTI combined with BMI in predicting AP and can be used as a simple and important auxiliary tool in the early clinical diagnosis of AP.

The present study possesses multiple advantages. It presents the inaugural analysis on the association of three novel visceral fat indices, CMI, LAP, and WTI, with the risk of AP. Second, the research incorporated many confounding factors that are known to be related to AP, including smoking and alcohol usage, exercise, cholelithiasis, hypertension, diabetes, and educational factors. Finally, the conclusion of this study may be regarded as having a satisfactory level of reliability because it is based on the data analysis of a large sample.

In addition, we are aware that this particular study possesses certain restrictions. In the beginning, participants were chosen from the populations of Chinese adults who were participating in the health checkup. Therefore, it is possible that the conclusions do not apply to people of diverse racial or ethnic backgrounds. Second,because all individuals were recruited from the same urban area and had lifestyles and eating habits that were comparable to one another, our ability to study more in-depth linkages or discoveries was constrained. Third, an issue that frequently arises in observational studies is the presence of unmeasured confounding variables. Even though we made adjustments to a large number of prospective confounding factors, we still did not find out the potentiality of residual confounding that was produced by factors that weren't assessed or identified.

## Conclusions

Finally, CMI, LAP, and WTI exhibited a positive and independent correlation with the risk of AP, and CMI showed a robust association. Significant and clinically efficient indicators of CMI, LAP, and WTI provide an easy way to identify people at higher risk of AP early. The clinical utility and convenience of CMI make it a promising tool for the prediction and prevention of AP.

### Supplementary Information

**Additional file 1: Fig. S1.** Flow chart of study population. **Figure S2.** The cumulative incidence rates in the quartile groups of LAP level. **Figure S3.** The cumulative incidence rates in the quartile groups of WTI level. **Figure S4.** The cumulative incidence rates in different groups of BMI. **Table S1.** Baseline characteristics of participants according to quartiles of LAP. **Table S2.** Baseline characteristics of participants according to quartiles of WTI. **Table S3.** Baseline characteristics of participants according to different groups of BMI.

## Data Availability

The datasets used and/or analyzed during the current study are available from the corresponding author on reasonable request.
