# Supplementary material for: Cardiometabolic Index (CMI), Lipid Accumulation Products (LAP), Waist Triglyceride Index (WTI) and the risk of acute pancreatitis: a prospective study in adults of North China
Source: Lipids Health Dis. 2023 Nov 9;22:190. doi: 10.1186/s12944-023-01948-3 (PMC10633920; doi:10.1186/s12944-023-01948-3)
Supplement: Supplementary file 1 — Additional file 1: Fig. S1. Flow chart of study population. Figure S2. The cumulative incidence rates in the quartile groups of LAP level. Figure S3. The cumulative incidence rates in the quartile groups of WTI level. Figure S4. The cumulative incidence rates in different groups of BMI. Table S1. Baseline characteristics of participants according to quartiles of LAP. Table S2. Baseline characteristics of participants according to quartiles of WTI. Table S3. Baseline characteristics of participants according to different groups of BMI. [file 12944_2023_1948_MOESM1_ESM.docx]

**Supporting Information**

**Fig. S1** Flow chart of study population.


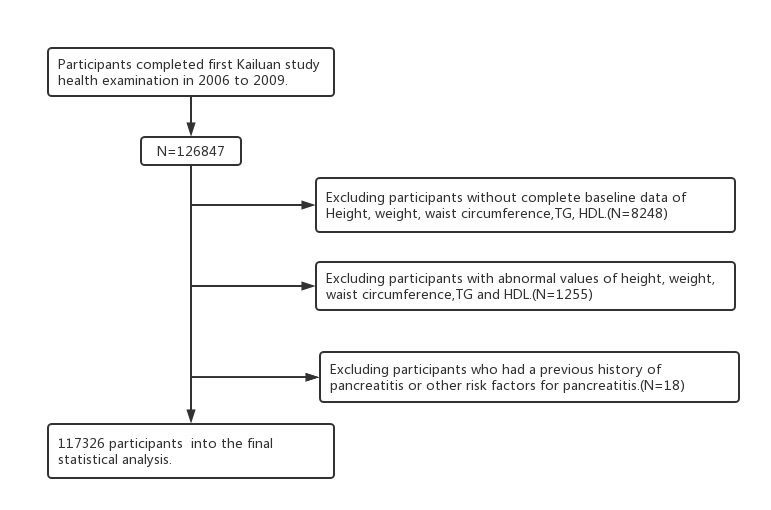


**Figure S2. The cumulative incidence rates in the quartile groups of LAP level**


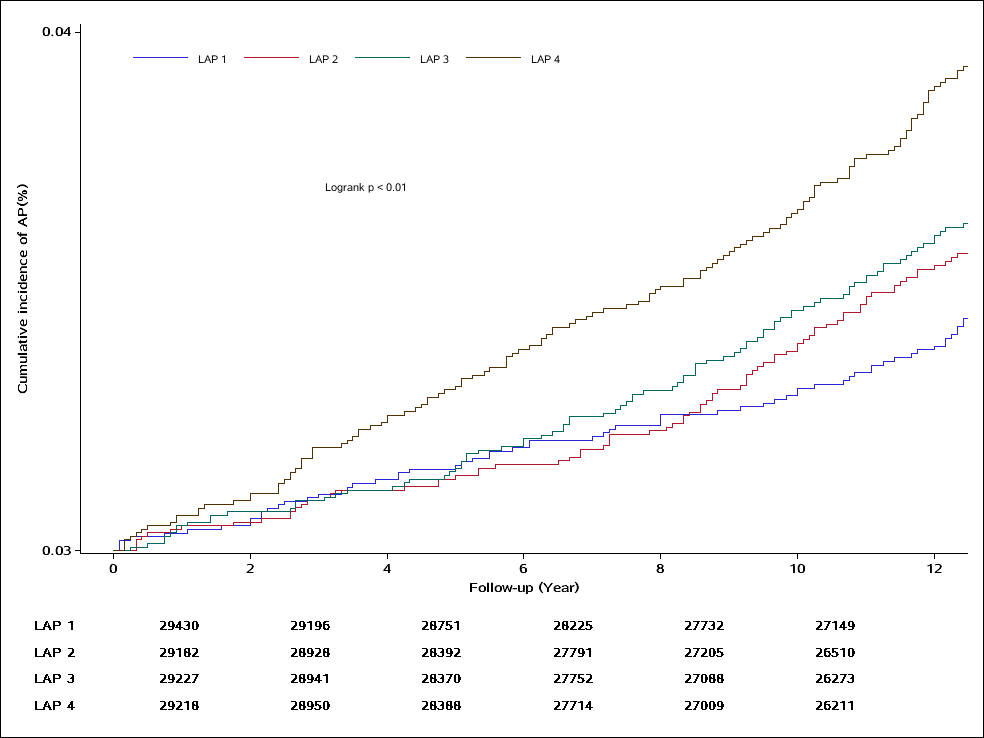


**Figure S3. The cumulative incidence rates in the quartile groups of WTI level**


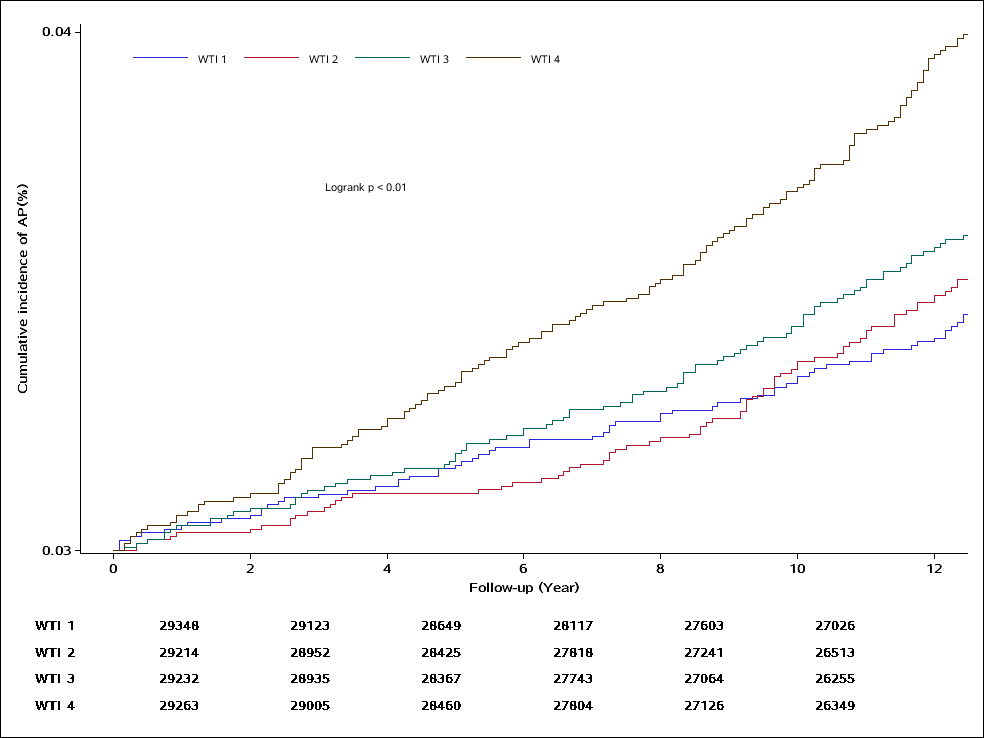


**Figure S4. The cumulative incidence rates in different groups of BMI**


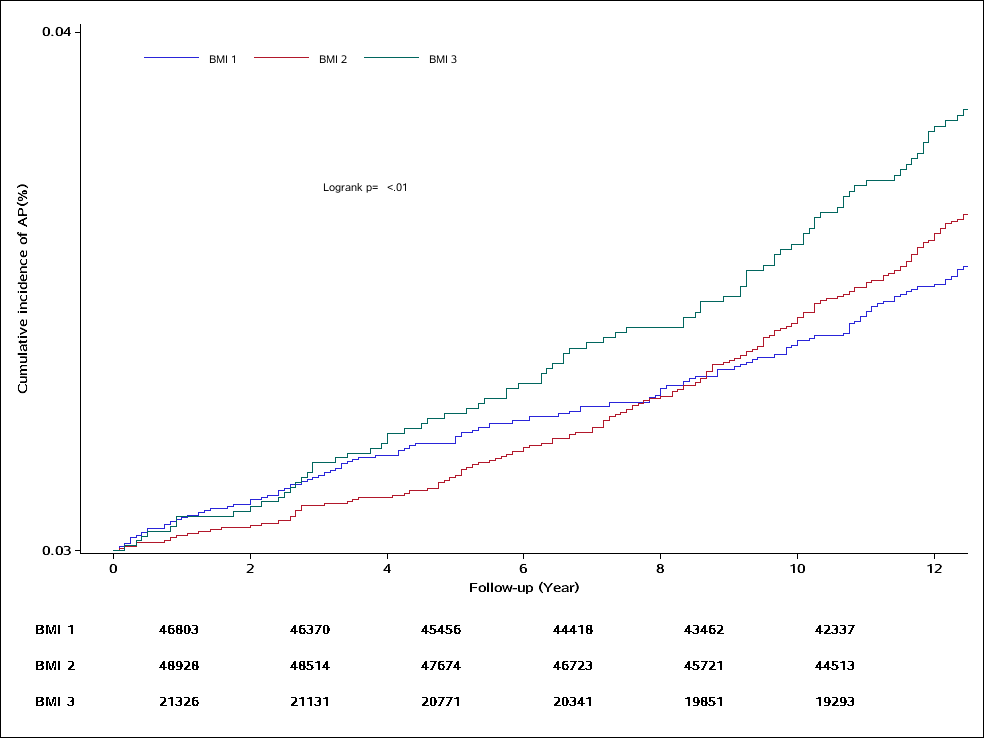


**Table S1.Baseline characteristics of participants according to quartiles of LAP**

| Characteristics | Quartiles of LAP | | | | *P* Value |
| --- | --- | --- | --- | --- | --- |
|  | LAP1(≤16.49) | LAP2(16.49-28.77) | LAP3(28.77-50.38) | LAP4(≥50.38) |  |
| Number | 29495 | 29250 | 29293 | 29288 |  |
| No. Of AP patient | 70 | 87 | 97 | 147 |  |
| Age,year | 48.23±14.08 | 51.34±12.64 | 52.58±12.30 | 52.40±11.59 | <0.001 |
| Men,n(%) | 23214 (78.70) | 23729 (81.12) | 23606 (80.59) | 23843 (81.41) | <0.001 |
| BMI,kg/m2 | 22.37±2.70 | 24.49±2.74 | 25.89±2.95 | 27.27±3.27 | <0.001 |
| WC,cm | 77.40±6.76 | 85.41±6.48 | 90.07±7.25 | 94.68±8.38 | <0.001 |
| Ht,cm | 166.99±6.74 | 167.69±6.72 | 167.84±6.90 | 168.18±7.06 | <0.001 |
| FBG,mmol/L | 5.18±1.29 | 5.35±1.43 | 5.54±1.66 | 5.91±2.14 | <0.001 |
| TG,mmol/L | 0.80±0.35 | 1.11±0.37 | 1.54±0.51 | 3.15±1.73 | <0.001 |
| TC,mmol/L | 4.67±0.95 | 4.91±0.96 | 5.07±1.10 | 5.11±1.46 | <0.001 |
| HDL-C,mmol/L | 1.55±0.38 | 1.53±0.38 | 1.51±0.38 | 1.49±0.40 | <0.001 |
| LDL-C,mmol/L | 2.36±0.88 | 2.45±0.93 | 2.46±0.94 | 2.35±1.07 | <0.001 |
| Drinker, n(%) | 12831 (43.50) | 11722 (40.08) | 12172 (41.55) | 12863 (43.92) | <0.001 |
| Smoker,n(%) | 12411 (42.08) | 11496 (39.30) | 11956 (40.82) | 12781 (43.64) | <0.001 |
| Physical exercise,n(%) | 4889 (16.58) | 4682 (16.01) | 4887 (16.68) | 4611 (15.74) | <0.001 |
| Hypertension, n (%) | 8034 (27.24) | 12062 (41.24) | 14512 (49.54) | 17056 (58.24) | <0.001 |
| Diabetes, n(%) | 1190 (4.03) | 2002 (6.84) | 2956 (10.09) | 4634 (15.82) | <0.001 |
| Cholelithiasis，n(%) | 721 (2.44) | 945 (3.23) | 1081 (3.69) | 1132 (3.87) | <0.001 |
| Education≥9 years,n(%) | 8476 (28.74) | 5801 (19.83) | 5771 (19.70) | 5632 (19.23) | <0.001 |
| Using lipid lowering drugs,n(%) | 115  (0.39) | 219  (0.75) | 331  (1.13) | 601  (2.05) | <0.001 |

**Table S2.Baseline characteristics of participants according to quartiles of WTI**

| Characteristics | Quartiles of WTI | | | | *P* Value |
| --- | --- | --- | --- | --- | --- |
|  | WTI 1(≤74.40) | WTI2(74.40-109.65) | WTI3(109.65-171.69) | LAP4(≥171.69) |  |
| Number | 29406 | 29280 | 29308 | 29332 |  |
| No. Of AP patient | 70 | 79 | 97 | 155 |  |
| Age,year | 49.10±13.87 | 51.37±12.91 | 52.46±12.47 | 51.60±11.62 | <0.001 |
| Men,n(%) | 21555 (73.30) | 23608 (80.63) | 24114 (82.28) | 25115 (85.62) | <0.001 |
| BMI,kg/m2 | 23.01±3.03 | 24.54±3.09 | 25.72±3.18 | 26.73±3.29 | <0.001 |
| WC,cm | 80.82±8.82 | 85.53±8.47 | 89.13±8.62 | 92.03±8.92 | <0.001 |
| Ht,cm | 166.83±6.91 | 167.61±6.80 | 167.90±6.86 | 168.35±6.82 | <0.001 |
| FBG,mmol/L | 5.16±1.25 | 5.36±1.44 | 5.57±1.69 | 5.89±2.14 | <0.001 |
| TG,mmol/L | 0.69±0.16 | 1.08±0.15 | 1.54±0.23 | 3.29±1.65 | <0.001 |
| TC,mmol/L | 4.63±0.93 | 4.91±0.94 | 5.12±1.15 | 5.10±1.44 | <0.001 |
| HDL-C,mmol/L | 1.55±0.39 | 1.54±0.38 | 1.51±0.38 | 1.49±0.40 | <0.001 |
| LDL-C,mmol/L | 2.30±0.92 | 2.46±0.94 | 2.50±0.91 | 2.36±1.05 | <0.001 |
| Drinker, n(%) | 12084 (41.09) | 11512 (39.32) | 12350 (42.14) | 13642 (46.51) | <0.001 |
| Smoker,n(%) | 11479 (39.04) | 11342 (38.74) | 12261 (41.83) | 13562 (46.24) | <0.001 |
| Physical exercise,n(%) | 4891 (16.63) | 4692 (16.02) | 4925 (16.80) | 4561 (15.55) | <0.001 |
| Hypertension, n (%) | 8347 (28.39) | 12193 (41.64) | 14498 (49.47) | 16626 (56.68) | <0.001 |
| Diabetes, n(%) | 1262 (4.29) | 2029 (6.93) | 3037 (10.36) | 4454 (15.18) | <0.001 |
| Cholelithiasis，n(%) | 835 (2.84) | 929 (3.17) | 1116 (3.81) | 999 (3.41) | <0.001 |
| Education≥9 years,n(%) | 8068 (27.44) | 5833 (19.92) | 5905 (20.15) | 5874 (20.03) | <0.001 |
| Using lipid lowering drugs,n(%) | 135  (0.46) | 211  (0.72) | 359  (1.22) | 561  (1.91) | <0.001 |

**Table S3.Baseline characteristics of participants according to different groups of BMI**

| Characteristics | BMI 1(<24) | BMI 2(24-28) | BMI 3(≥28) | *P* Value |
| --- | --- | --- | --- | --- |
| Number | 46919 | 49026 | 21381 |  |
| No. Of AP patient | 136 | 163 | 102 |  |
| Age,year | 50.38±13.87 | 51.86±11.88 | 51.11±12.32 | <0.001 |
| Men,n(%) | 35984 (76.69) | 40897 (83.42) | 17511 (81.90) | <0.001 |
| WC,cm | 80.87±8.29 | 88.66±7.25 | 95.96±8.52 | <0.001 |
| Ht,cm | 167.45±6.86 | 167.93±6.77 | 167.58±7.10 | <0.001 |
| FBG,mmol/L | 5.30±1.58 | 5.58±1.73 | 5.73±1.76 | <0.001 |
| TG,mmol/L | 1.32±1.07 | 1.77±1.36 | 2.08±1.45 | <0.001 |
| TC,mmol/L | 4.83±1.07 | 5.00±1.19 | 5.04±1.21 | <0.001 |
| HDL-C,mmol/L | 1.57±0.40 | 1.50±0.38 | 1.47±0.37 | <0.001 |
| LDL-C,mmol/L | 2.33±1.01 | 2.45±0.92 | 2.47±0.93 | <0.001 |
| Drinker, n(%) | 19200 (40.92) | 21385 (43.62) | 9003 (42.11) | <0.001 |
| Smoker,n(%) | 19134 (40.78) | 20789 (42.40) | 8721 (40.79) | <0.001 |
| Physical exercise,n(%) | 7332 (15.63) | 8174 (16.67) | 3563 (16.66) | <0.001 |
| Hypertension, n (%) | 14864 (31.68) | 23650 (48.24) | 13150 (61.50) | <0.001 |
| Diabetes, n(%) | 2823 (6.02) | 5120 (10.44) | 2839 (13.28) | <0.001 |
| Cholelithiasis，n(%) | 1239 (2.64) | 1759 (3.59) | 881 (4.12) | <0.001 |
| Education≥9 years,n(%) | 11299 (24.08) | 9939 (20.27) | 4442 (20.78) | <0.001 |
| Using lipid lowering drugs,n(%) | 286  (0.61) | 588  (1.20) | 392  (1.83) | <0.001 |

Abbreviations:AP=acute pancreatitis,CMI=Cardiometabolic Index;LAP=Lipid Accumulation Products;WTI=Waist Triglyceride Index ;BMI=body mass indes;WC=Waist circumference;Ht=Height;FBG=fasting blood glucose;TC=total cholesterol, TG=triglyceride, HDL=high-density lipoprotein, LDL=low-density lipoprotein.
